# Economic empowerment of free trade zone identity labels: the impact of multi-dimensional identity labels on consumer purchase intention for organic food

**DOI:** 10.3389/fnut.2025.1681453

**Published:** 2026-01-08

**Authors:** Yan Zhang

**Affiliations:** 1 International Business School, Qingdao Huanghai University, Qingdao, China; 2 School of Economics and Management, China University of Petroleum (East China), Qingdao, China

**Keywords:** free trade zone, identity label, organic food, purchase intention, regional identity, regional attitude

## Abstract

Extensive prior research has confirmed that label information on food packaging is a crucial factor in stimulating consumer purchase intention, such as labels indicating sugar content, fat content, and purine content. However, few studies have focused on the impact of multi-dimensional geographical identity labels for organic food on consumer purchase intention. This study takes the geographical identity of organic food using free trade zones and provincial administrative regions as examples, treating them as dual identities of organic food production areas. We recruited 848 participants and conducted three online scenario-based experiments to verify the impact of multi-dimensional (vs. single) identity labels of organic food production areas on consumer purchase intention. The results show that compared to single identity labels, multi-dimensional identity labels of organic food production areas can effectively enhance consumer purchase intention. Among these, the regional identity triggered by multi-dimensional identity labels becomes an important factor in boosting consumer purchase intention. However, consumer regional attitudes can effectively moderate the relationship between multi-dimensional identity labels and consumer purchase intention. This study is the first to verify the positive role of multi-dimensional identity labels of organic food production areas in the field of organic food consumption, providing valuable measures for the marketing of organic food in free trade zones. While these findings are derived from the context of Chinese free trade zones and may be influenced by cultural and economic specificities, they offer a foundation for future cross-cultural validations in global organic food markets.

## 1 Introduction

Driven by increasing consumer awareness of health, environmental sustainability, and food safety, organic food has become an essential part of the global food market (1–4). Unlike conventional agriculture, organic farming avoids synthetic pesticides and chemical fertilizers, promoting ecological balance and biodiversity conservation (5). Consistent research indicates that compared to conventionally grown counterparts, organic food may offer better nutritional profiles, such as higher levels of antioxidants and lower pesticide residues (6–8). Despite the advantages of organic food, its market share is still relatively small. Specifically, in the European Union, North America, Japan and other regions, the share of organic food in the local food market is only 2.5–3.5% (9). This limited market share is often attributed to higher price points and inadequate consumer education (10, 11), highlighting the necessity of investigating factors influencing organic food purchases, particularly the role of labeling strategies in shaping consumer behavior. Food labels, as a key tool for conveying basic information and shaping consumer perceptions, play a critical role in bridging the gap between producers and buyers (12–14).

Consumer perception and purchase intention for organic food are influenced by the complex interplay of multiple factors, among which food labeling is a key determinant (15, 16). Research has shown that labels, including nutritional declarations, geographical indications, purine content, and organic certifications, significantly influence consumer decision-making processes (17–19). Especially for organic food, certifications and geographical labels are paramount, as they provide guarantees of authenticity and traceability—attributes highly valued by health-conscious and environmentally aware consumers (20). However, while the impact of single-attribute labels has been extensively studied, the effects of multi-dimensional or composite identity labels remain underexplored. This research gap is particularly relevant to organic food, where the interplay of multiple geographical identities may enhance consumer appeal and thereby strengthen purchase intention.

Identity labels associate food with specific geographical, cultural, or social entities (21, 22). They bridge the information gap between producers and consumers, becoming an important marketing strategy (23, 24). For organic food, trust and authenticity in the production process are paramount, and identity labels tied to geographical origin can enhance perceived value and credibility (25, 26). In recent years, the rise of free trade zones has introduced a new dimension to regional identity labels. Free trade zones are specialized economic areas established by governments to stimulate trade and investment, with incentives including tax exemptions, simplified customs procedures, and enhanced market access (27, 28). These zones not only promote international trade but also support domestic industrial development by reducing operational costs and improving supply chain efficiency (29). While single identity labels can influence consumer behavior, they may not fully satisfy the multifaceted information needs of today’s consumers. Multi-dimensional identity labels combine various identifiers, providing richer narratives about food origins and attributes (30). For organic food, consumers prioritize transparency regarding production sources and environmental conditions, and such labels can enhance trust and appeal (31). By offering a more comprehensive identity framework, these labels can strengthen consumer confidence and preferences in an increasingly crowded market. Consequently, organic foods with dual identities are more likely to stimulate consumer purchase intention compared to those with single identities.

This study investigates the impact of multi-dimensional identity labels on consumer purchase intention for organic food, using free trade zones and provincial administrative regions as examples of geographical identity labels for organic food production areas. Based on social identity theory, we hypothesize that multi-identity labels enhance purchase intention by fostering regional identity alignment, where consumer affinity for specific regions amplifies the attractiveness of the food. Empirical evidence from three scenario-based experiments involving 848 participants demonstrates that multi-identity labels outperform single-identity labels in driving purchase intention, with regional identity serving as a key mediator. Furthermore, consumer regional attitudes moderate this relationship, indicating an interaction between label design and consumer psychology. The significance of this study lies in its dual contribution to organic food marketing and free trade zone economic strategies. By demonstrating the positive impact of multiple identity labels, this research provides actionable insights for marketers seeking to differentiate organic food and enhance consumer appeal. The findings are expected to inform industry practitioners and policymakers, promoting sustainable growth in the organic food sector and strengthening the economic vitality of free trade zones.

## 2 Literature review and theoretical deduction

### 2.1 Social identity theory

Social identity theory posits that an individual’s self-concept is partially derived from their membership in specific social groups (32). These group memberships collectively constitute the individual’s social identity. Social identity not only shapes self-perception but also influences attitudes and behavioral choices through intergroup comparisons (33, 34). The core mechanisms include in-group favoritism and out-group discrimination, with the former manifesting as support for one’s own group and the latter as exclusion of other groups (35, 36). These mechanisms stem from the psychological need to enhance self-worth by elevating the status of one’s own group (37). This phenomenon is particularly pronounced in the consumer domain. When consumers associate themselves with a specific region, the regional identity label becomes part of their social identity (38). For instance, if consumers identify with their local identity, foods labeled with local identities may be more preferred because they reinforce this identity.

In consumer behavior research, social identity theory has been widely applied to explain preferences for foods or brands (39). Group norms and group cohesion are key variables influencing individual behavior (40). Group norms define the behavioral guidelines that members are expected to follow, while group cohesion strengthens individuals’ adherence to these norms (41, 42). For example, within an environmentalist group, purchasing organic food may become a normative behavior, with individuals exhibiting higher purchase intention due to group pressure or a sense of belonging (43). Social identity theory also reveals the role of identity salience, where individuals are more likely to act according to a particular identity when it is activated (44, 45). Multi-dimensional identity labels for organic food production areas, such as the simultaneous use of free trade zone and provincial administrative region labels, provide consumers with richer social identity cues. Such multi-dimensional labels may enhance consumers’ sense of identification and trust by activating multiple social identities. For example, a free trade zone identity may be associated with innovation and high quality, while a provincial administrative region identity may evoke cultural identification (46, 47). The combination of these identities may produce a synergistic effect, amplifying consumers’ positive reactions. Existing research confirms that in food purchasing decisions, consumers tend to choose foods that reflect their social identity and self-concept (48, 49). This implies that multi-dimensional identity labels for organic food production areas can influence purchase intention by activating consumers’ social identity. Internationally, studies on local food groups show how geographical labels activate place-based identities, linking personal values to collective sustainability efforts and enhancing purchase behaviors (50). Based on social identity theory, this study proposes that multi-dimensional identity labels enhance purchase intention by activating consumers’ social identities, thereby significantly improving the purchase intention for organic foods.

Additionally, recent interdisciplinary work on healthy food perception offers complementary insights into the psychological processes underlying identity activation and symbolic cues. For instance, research examining the healthy eating movement on social media demonstrates how health-related signals and social cues influence self-perceptions, leading to internalized ideals that distort body shape perception and trigger maladaptive behavioral responses, such as restrictive or emotional eating (51). While centered on digital platforms, this framework parallels the symbolic interpretation of multi-dimensional labels in organic food, where geographical and institutional cues may intersect with aspirational branding to shape consumers’ health-conscious identities, enhancing cognitive alignment and purchase motivations in contexts where social identity and self-concept converge.

### 2.2 Multiple social categorization theory

While social identity theory elucidates how group membership shapes self-concept, it does not fully address the cognitive dynamics when individuals simultaneously activate multiple group identities. The theory of multiple social categorization posits that individuals hold complex social identities comprising intersecting categories (e.g., profession, nationality, regional affiliation) (52). When multiple identities are salient, they may exhibit additive or synergistic effects on attitudes and behaviors, depending on their compatibility (53).

In the context of geographical labels, multi-dimensional identity labeling (e.g., “Free Trade Zone + Provincial Region”) activates two distinct yet complementary social categories: Economic-Administrative Identity (e.g., Free Trade Zone as a symbol of innovation, quality standards, and global trade) and Cultural-Territorial Identity (e.g., Provincial region evoking tradition, local heritage, and ecological authenticity). Such labeling leverages the complexity advantage: Consumers perceive products with multi-dimensional identities as richer in symbolic value, enhancing trust and appeal through cross-category reinforcement. This provides a theoretical micro-foundation for why multi-dimensional labels outperform single-identity labels. Empirically, studies on Free Trade Zones in China demonstrate that they serve as identity markers by associating with high regulatory standards and international prestige, fostering consumer pride and loyalty (54). Conceptually, Free Trade Zones function as identity markers because they embody aspirational economic modernity, allowing consumers to align their self-concept with globalized, innovative groups, thereby differentiating from traditional single-region identities. This synergy is particularly potent in organic food, where Free Trade Zones labels signal enhanced traceability and sustainability, complementing provincial cultural ties. Globally, research on multidimensional front-of-pack labels integrating nutrition, environment, and processing shows similar synergies, improving consumer choices across Europe and the Americas by addressing non-collinear dimensions (55).

### 2.3 Multi-dimensional geographical identity labels of organic food production areas and consumer purchase intention

Multi-dimensional geographical identity labels for organic food production areas refer to the practice of marking multiple identities from the same region on food packaging. This labeling design provides consumers with richer information, potentially influencing their perceptions of food quality, safety, and environmental sustainability (13, 56). Research indicates that geographical information is a critical cue in consumer purchasing decisions, particularly in the organic food domain, where consumers often associate production origin with credibility and food value (57, 58). Multi-dimensional identity labels integrate the symbolic meanings of different regional identities, potentially enhancing consumer trust and preference. For example, a free trade zone label may imply internationalization and high standards, while a provincial administrative region label may evoke regional cultural identification (59, 60). The combination of these identities may increase the appeal of the food, thereby boosting purchase intention.

Building on multiple social categorization theory, we argue that multi-dimensional identity labels create a compound regional narrative. For instance, “Hainan Free Trade Zone + Hainan Province” merges the Free Trade Zone association with regulatory rigor and international access with the province’s image of tropical biodiversity and cultural distinctiveness. This synergy amplifies perceived authenticity and value beyond what either label achieves alone. Empirically, Free Trade Zones enhance identity salience by linking to economic empowerment and global competitiveness, as evidenced in studies showing increased consumer preference for Free Trade Zone-sourced goods due to perceived prestige (35, 61). Conceptually, Free Trade Zones act as identity markers by fulfilling consumers’ needs for social distinction, where affiliation with an Free Trade Zone signals membership in a progressive, high-status group, thereby elevating purchase intention for organic products. A systematic review of international research confirms origin labels substantially influence choices worldwide, with preferences for local/domestic over imported, moderated by quality cues like organic certifications (62). Multi-labeling studies further show that combining valences (positive/negative) can backfire via confusion but enhance intentions when synergistic (63).

Based on the above analysis, we propose the following hypothesis:

*H*1: Compared to single identity labels, consumers exhibit higher purchase intention for organic food products with multi-dimensional identity labels.

### 2.4 The mediating effect of regional identity

Regional identity refers to an individual’s sense of belonging and identification with a specific region, typically stemming from birthplace, residence, or cultural background (61, 64). In consumer behavior, regional identity is considered a key driver of preferences for region-specific foods (65, 66). Research shows that the stronger consumers’ sense of identification with a particular region, the greater their trust and purchase intention for foods from that region (67). In the organic food domain, production origin labels may influence consumer purchasing behavior by activating regional identity. For example, when a food product is labeled as “Guangdong Free Trade Zone + Guangdong Province,” residents of Guangdong may develop a favorable attitude toward the product due to regional identification, thereby increasing their purchase intention.

Multi-dimensional identity labels, by integrating multiple regional identities, may more effectively activate consumers’ regional identity. A free trade zone identity may be associated with modernization and economic vibrancy, while a provincial administrative region identity may evoke historical culture and tradition (60). The combination of these identities may strengthen consumers’ sense of identification. This sense of identification could translate into positive evaluations of the food, such as perceiving it as more aligned with personal values or social expectations, ultimately increasing purchase intention. Existing studies support the mediating role of identity, such as cases where consumers prefer domestic products due to national identity (68). Multi-dimensional identity labels strengthen regional identity not merely through singular group affiliation, but by integrating complementary identities into a cohesive self-concept. When consumers internalize both the economic prestige of an Free Trade Zone and the cultural resonance of their province, their sense of belonging becomes multifaceted and self-reinforcing. This enriched identity mediates the label–purchase intention link. Meta-analyses from 11 countries show labeling reduces unhealthy intakes by 6–13%, with identity-driven preferences mediating choices in diverse contexts (69).

Based on the above analysis, we propose the following hypothesis:

*H*2: Regional identity has a significant mediating effect on the relationship between multi-dimensional identity labels of organic food production areas and consumer purchase intention.

### 2.5 The moderating effect of regional attitude

Regional attitude refers to consumers’ overall evaluation and emotional inclination toward a specific region, influenced by factors such as regional culture, economic development, and media coverage (49, 70). Research indicates that when consumers hold a positive attitude toward a particular region, their evaluations of foods from that region are higher, and vice versa (71, 72). Therefore, the effects of multi-dimensional identity labels may vary depending on consumers’ attitudes toward free trade zones or provincial administrative regions. More importantly, regional attitudes influence how consumers interpret and trust the information conveyed by multi-dimensional identity labels. A positive regional attitude makes consumers more likely to trust and accept the advantages and features claimed by foods with such labels (73). Conversely, a negative regional attitude may lead consumers to question and distrust the food information. For instance, if consumers perceive a region as having lax regulation or inconsistent food quality, they may reduce their purchase intention, even if the multi-dimensional identity label is theoretically appealing (74). Thus, regional attitudes play a crucial role in regulating the relationship between multi-dimensional identity labels and consumer purchase intention by influencing information processing and trust mechanisms.

Based on the above analysis, we propose the following hypothesis:

*H*3: Regional attitudes significantly moderate the relationship between multi-dimensional identity labels of organic food production areas and consumer purchase intention.

## 3 Study overview

To further validate the three propositions outlined above, this study conducted three online scenario-based experiments. Online scenario-based experiments emphasize conducting experimental operations and observations in settings that approximate real-world or specific contexts to investigate the behavior, cognition, attitudes, or decision-making of individuals or groups under the influence of environmental, contextual, or socio-cultural factors. Specifically, in Experiment 1, we analyzed the impact of multi-dimensional (vs. single) identity labels of organic food production areas on consumer purchase intention, testing Hypothesis H1. In Experiment 2, we explored the mediating role of regional identity in the relationship between multi-dimensional (vs. single) identity labels and consumer purchase intention, testing Hypothesis H2. In Experiment 3, we examined the moderating role of regional attitudes in the relationship between multi-dimensional (vs. single) identity labels and consumer purchase intention, testing Hypothesis H3. To enhance experimental accuracy, we used “Xinjiang Uyghur Autonomous Region Free Trade Zone” and a regional outline of Xinjiang Uyghur Autonomous Region as stimuli in Experiment 1; “Hainan Free Trade Zone” and a regional outline of Hainan Province as stimuli in Experiment 2; and “Henan Free Trade Zone in China” and a regional outline of Henan Province as stimuli in Experiment 3. The demographic information of the three experiments is presented in Table 1; the study framework is shown in Table 2; and all measurement items are listed in Appendix A.

**Table 1:** Demographic characteristics of participants across the three experiments.

Variables	Items	Experiment 1		Experiment 2		Experiment 3	
		Frequency	Proportion	Frequency	Proportion	Frequency	Proportion
Gender	Male	137	52.6%	140	49.3%	142	50.4%
	Female	145	47.4%	144	50.7%	140	49.6%
Age	18–25 years old	68	24.1%	90	25.4%	77	27.3%
	26–40 years old	80	28.4%	133	46.9%	83	29.4%
	41–60 years old	59	20.9%	29	10.2%	61	21.6%
	61 years old and above	75	26.6%	32	11.3%	61	21.6%
Educational background	Primary school	44	15.6%	36	12.7%	39	13.8%
	Junior high school	48	17.0%	40	14.1%	39	13.8%
	Technical secondary school/ Senior high school	45	16.0%	51	18.0%	39	13.8%
	Junior college	71	25.2%	52	18.3%	77	27.3%
	Undergraduate college	57	20.2%	44	15.5%	75	26.6%
	Postgraduate	17	6.0%	61	21.4%	13	4.6%

This table summarizes key participant demographics, including age, gender, and education level, for each experiment (*N* = 848 total). Data were collected via the Credamo platform to ensure a diverse sample representative of Chinese consumers.

**Table 2:** Experimental framework of the three studies.

Experiment	Experiment 1	Experiment 2	Experiment 3
Purpose	To test for main effects (H1)	To test the mediating effect of regional identity (H2)	To test the modulation effect of regional attitude(H3)
Independent variable	Multi-dimensional (vs. single) identity labels	Multi-dimensional (vs. single) identity labels	Multi-dimensional (vs. single) identity labels
Dependent variable	Purchase intention	Purchase intention	Purchase intention
Mediators	-	Regional identity	-
Moderator	-	-	Regional attitude
Methods	ANOVA	ANOVA	ANOVA
		PROCESS 4	PROCESS 1
Results	Supported H1	Supported H2	Supported H3

This table outlines the design, objectives, stimuli, and hypotheses tested in each experiment, providing a comprehensive overview of the study’s structure.

Regions were selected for diversity: Xinjiang (Experiment 1) for ethnic and remote representation; Hainan (Experiment 2) for tropical and tourism-oriented FTZ; Henan (Experiment 3) for central agricultural hub, ensuring generalizability across China’s FTZ landscape.

With respect to potential bias, we first used anonymization and confidentiality during the data collection phase to lower societal expectations. Immediately following this, in participant self-selection bias, we used ambiguous experimental titles and descriptions, thereby reducing participant sensitivity.

To minimize potential price-related biases, all participants were informed that the organic foods presented across all conditions were identical in price and quality, differing only in labeling design. This price-control approach aligns with prior labeling effect research focusing on perceptual and identity-based mechanisms (10, 19). By keeping the price constant, we ensured that observed variations in purchase intention were primarily attributable to the informational and symbolic dimensions of identity labels, rather than to economic considerations.

Regarding demographic variables such as age, gender, and education level, these were collected and reported in Table 1 to characterize the sample and ensure its diversity and representativeness of Chinese consumers. These variables were not tested as control variables or moderators in the primary analyses, as preliminary exploratory analyses showed no significant associations with purchase intention (all ps > 0.05) and did not alter the main findings. This decision focused the analyses on the core theoretical constructs (identity labels, regional identity, and regional attitudes) while minimizing model complexity and Type I error risk. However, gender was explicitly tested as a control variable in Experiment 1 (no significant effect), and mood was controlled in Experiment 2, as detailed in the respective sections.

## 4 Experiment 1: The multi-dimensional identity labels identity labels of organic food origin and consumers’ purchase intentions

### 4.1 Experimental design

In Experiment 1, we employed a single-factor between-subjects design (identity label: single vs. multiple) to examine the impact of multi-dimensional identity labels of organic food production areas on consumer purchase intention. Using a professional data collection platform, Credamo,^1^ we randomly recruited 300 participants. However, 18 participants were excluded due to incomplete questionnaires and high consistency in their responses, resulting in a final sample of 282. Demographic information is detailed in Table 1. All participants were assigned to a large mall as the marketing scenario and randomly divided into either the single identity group (*N* = 136) or the multi-dimensional identity group (*N* = 146).

**Figure 1:**
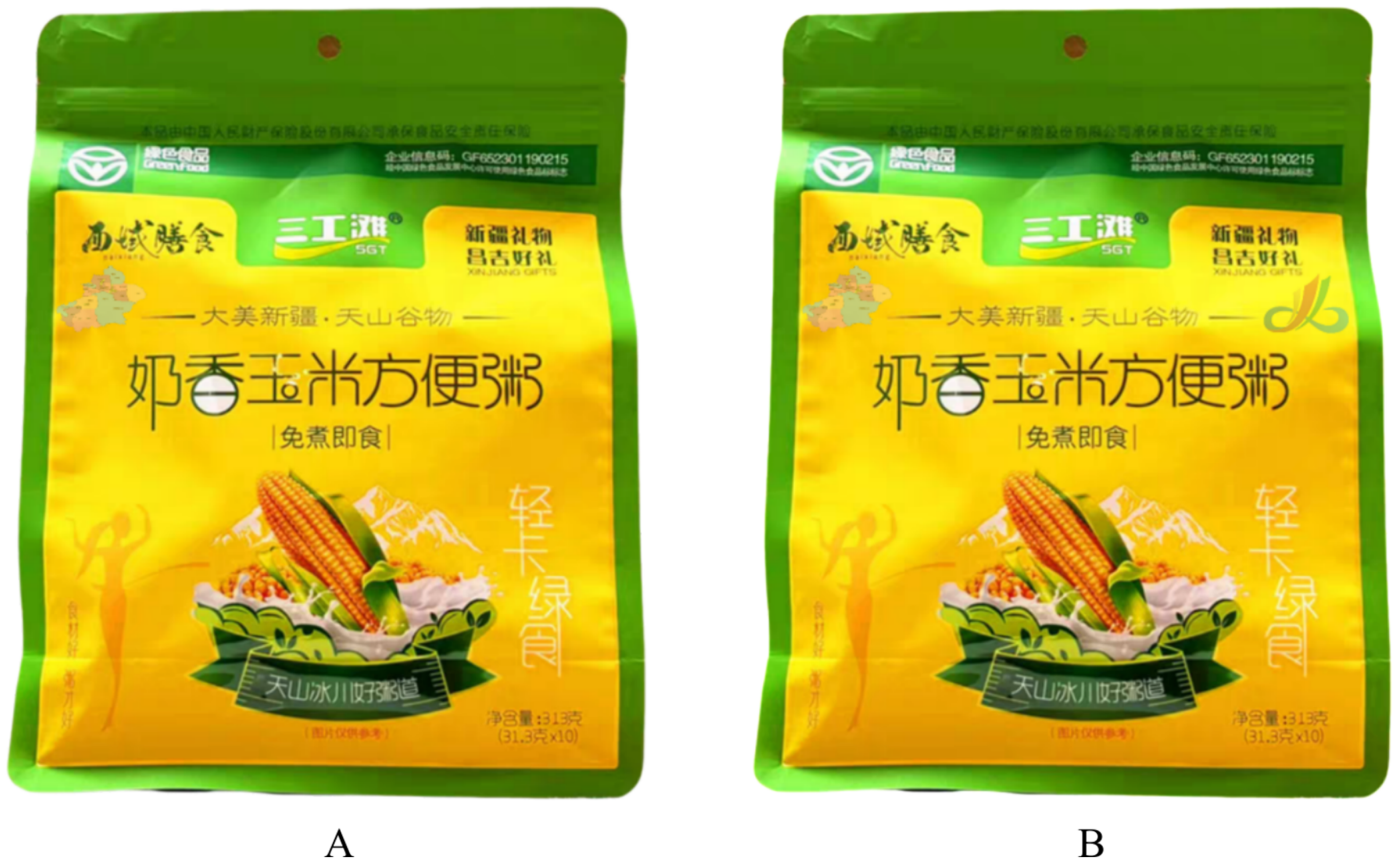
Stimulus materials used in experiment 1 to manipulate identity labels for organic food packaging. Panel **A** depicts the single identity label condition, featuring only the geographical outline of the Xinjiang Uyghur Autonomous Region on the packaging, representing a traditional regional identity. Panel **B** depicts the multi-dimensional identity label condition, featuring both the geographical outline of the Xinjiang Uyghur Autonomous Region and the designation of the Xinjiang Free Trade Zone, combining cultural-territorial and economic-administrative identities to test their impact on consumer perceptions.

Experimental Procedure: We instructed all participants to imagine they were purchasing organic food from Hainan in a large shopping mall. Subsequently, we provided all participants with an introduction to the organic food, as detailed in Appendix B-1. Participants in the multi-dimensional identity group were shown food advertisements featuring both the Xinjiang Uyghur Autonomous Region Free Trade Zone and a regional outline of Xinjiang; those in the single identity group were shown advertisements featuring only the regional outline of Xinjiang, as illustrated in Figure 1. To validate the manipulation, we asked participants about the complexity of the label information, for example, “The information about the production area of the organic food shown on the packaging gives you a feeling of including multiple different aspects or attributes?” (1 = Strongly Disagree, 7 = Strongly Agree). Following this, participants were asked about their purchase intention, for example, “After learning about the production area label of the aforementioned food, I am willing to purchase it?” (1 = Strongly Disagree, 7 = Strongly Agree) (17). Finally, participants provided necessary demographic information.

### 4.2 Research results

Manipulation Check: We used label information complexity as a test variable and conducted an independent samples t-test. The results indicated that organic foods with multi-dimensional identity labels (M = 5.62, SD = 1.602) were perceived as having higher information complexity compared to single identity labels (M = 5.04, SD = 1.857, *t* = 2.809, *p* = 0.008, Cohen d = 1.730), confirming the effectiveness of the manipulation in Experiment 1.

Main Effect Test: We used the identity label (single vs. multi-dimensional) as the independent variable and purchase intention as the dependent variable, employing a single-factor ANOVA. The results showed that consumers exhibited higher purchase intention for organic foods with multi-dimensional identity labels (M = 5.80, SD = 1.456) compared to those with single identity labels (M = 4.34, SD = 1.643, *F*(1,280) = 62.836, *p* < 0.001, η^2^ = 0.183), thereby verifying Hypothesis H1.

Control Variable Analysis: Given that previous research has found that males exhibit significantly higher purchase intention for organic foods than females, we included participant gender as a control variable and conducted a single-factor ANOVA. The results showed no significant impact of gender on purchase intention (*F*(1,280) = 0.301, *p* = 0.584). Thus, gender did not significantly influence the experimental results, further validating Hypothesis H1.

### 4.3 Interpretation of results

Experiment 1 validated that multi-dimensional identity labels of organic food production areas significantly influence consumer purchase intention (F(1,280) = 62.836, *p* < 0.001, η^2^ = 0.183). Multi-dimensional identity labels convey the value and unique qualities of organic food production areas across various dimensions. Specifically, these labels highlight the natural environmental advantages, traditional farming techniques, and cultural features of the production area, constructing a rich food image that arouses consumers’ curiosity and interest, thereby enhancing their recognition and purchase intention. This aligns with social identity theory, where multiple cues activate stronger in-group favoritism toward regionally tied products (75). Additionally, by controlling for gender, we strengthened the robustness of the experiment (no significant gender effect, *F*(1,280) = 0.301, *p* = 0.584).

Despite these findings, Experiment 1 did not further investigate whether a mediating factor exists between multi-dimensional identity labels and purchase intention. Therefore, in Experiment 2, we introduced regional identity as a mediator to examine its role in the relationship between multi-dimensional identity labels and purchase intention.

## 5 Experiment 2: The mediating role of regional identity

### 5.1 Experimental design

Experiment 2 aimed to analyze the mediating role of regional identity in the relationship between multi-dimensional identity labels and consumer purchase intention, using a single-factor between-subjects design (identity label: multi-dimensional vs. single). We recruited 300 participants using the professional data collection platform, Credamo (see Footnote 1), excluding 16 participants due to incomplete questionnaires or high response consistency, resulting in a final sample of 284. Demographic details are presented in Table 1. Participants were randomly assigned to either the single identity group (*N* = 140) or the multi-dimensional identity group (*N* = 144), with the marketing scenario set in a local organic food marketplace.

**Figure 2:**
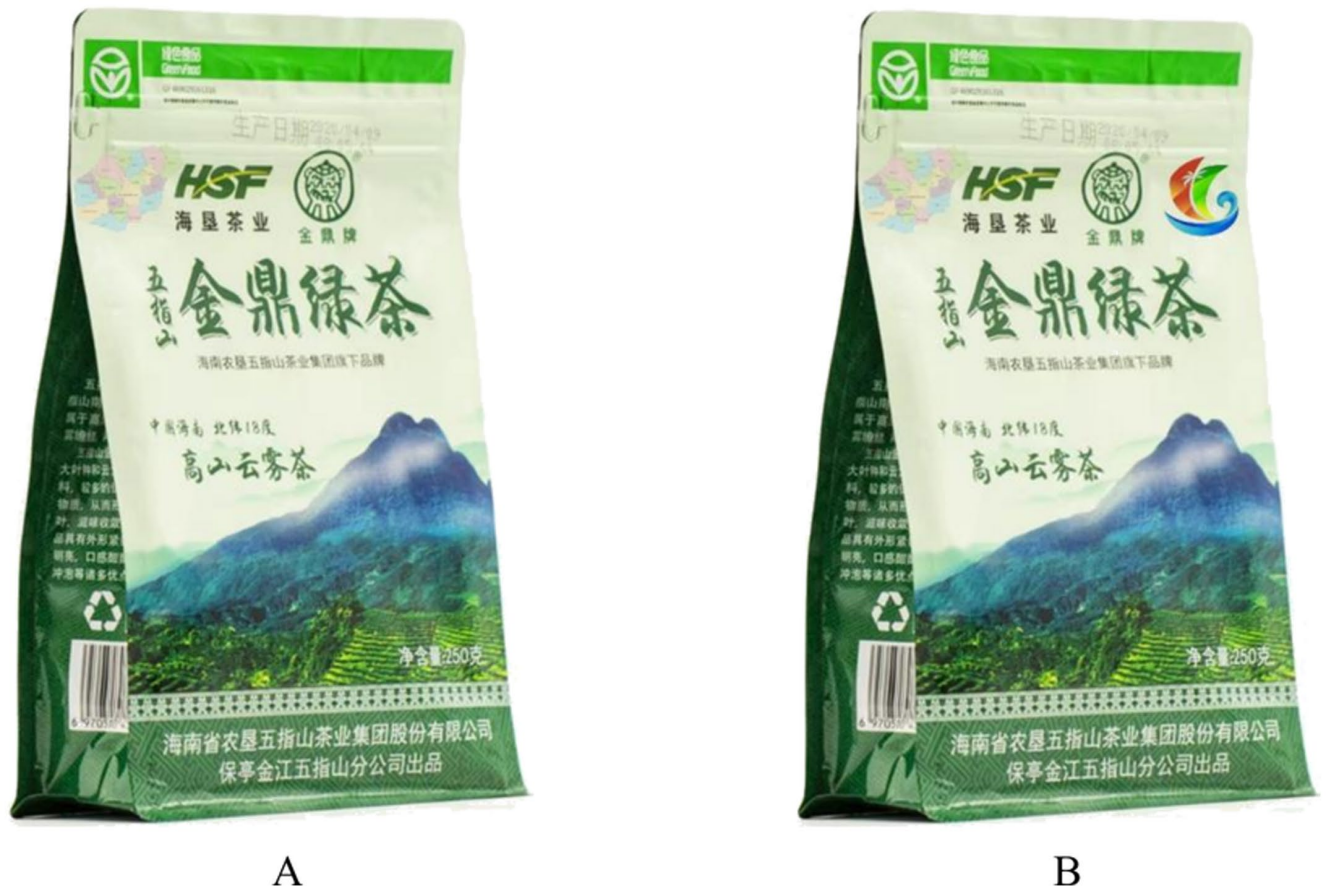
Stimulus materials used in experiment 2 to manipulate identity labels for organic food packaging. Panel **A** illustrates the single identity label condition with the outline of Hainan Province, emphasizing cultural and territorial aspects. Panel **B** illustrates the multi-dimensional identity label condition, incorporating both the Hainan Province outline and the Hainan Free Trade Zone label, to examine the synergistic effects on regional identity and purchase intention.

Experimental Procedure: Participants were instructed to imagine they were selecting organic food in a local marketplace. Subsequently, they were provided with an introduction to the organic food used in the experiment, as detailed in Appendix B-2. Participants in the multi-dimensional identity group were shown food advertisements featuring both the Hainan Free Trade Zone and a regional outline of Hainan; those in the single identity group were shown advertisements featuring only the Hainan regional outline, as illustrated in Figure 2. To check the manipulation, participants were asked about the diversity of label information, for example, “The description of Hainan as the production area emphasizes multiple identities or features?” Following this, participants responded to questions assessing regional identity, such as “I feel proud to be a member of this region” (1 = Strongly Disagree, 7 = Strongly Agree) (76) (Cronbach’s *α* = 0.966, χ^2^/df = 2.136, GFI = 0.931, AGFI = 0.880, RMSEA = 0.063, CFI = 0.983, TLI = 0.974).

Given that previous research has identified emotion as an important factor influencing purchase intention, validated in numerous studies, all participants also completed a emotion assessment. For example, “Do you agree that your current mood is positive (happy, pleasant, relaxed)?” (1 = Strongly Disagree, 7 = Strongly Agree) (77).

### 5.2 Research results

Manipulation Check: Using label information diversity as a test variable, we conducted an independent samples t-test. The results showed that organic foods with multi-dimensional identity labels (M = 4.921, SD = 1.870) were perceived as having higher information diversity compared to single identity labels (M = 3.784, SD = 1.833, *t* = 5.173, *p* < 0.001, Cohen d = 1.851), confirming the effectiveness of the manipulation in Experiment 2.

Main Effect Analysis: Identity label (multi-dimensional vs. single) was the independent variable, and purchase intention was the dependent variable, analyzed via single-factor ANOVA. The results indicated that purchase intention was significantly higher for multi-dimensional identity labels (M = 5.72, SD = 1.447) compared to single identity labels (M = 4.69, SD = 2.01, *F*(1,282) = 24.301, *p* < 0.001, η^2^ = 0.079), thereby validating Hypothesis H1.

**Figure 3:**
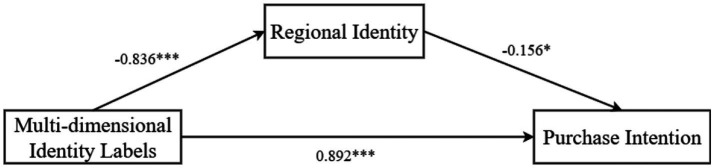
Path coefficient map illustrating the mediating effect of regional identity in the relationship between multi-dimensional identity labels and consumer purchase intention. **The model shows standardized beta coefficients for direct and indirect paths, with regional identity partially mediating the effect (*β* = 0.130, 95% CI = [0.009, 0.149]). Significance levels: ***p < 0.001, **p < 0.01, *p < 0.05. This figure is based on Process Model 4 analysis with 5,000 bootstraps.

Mediation Effect Test: We used Process Model 4 to examine the mediating role of regional identity (Bootstrap sample: 5000) (78), with identity label as the independent variable and purchase intention as the dependent variable. The results revealed that multi-dimensional identity labels significantly influenced purchase intention (*β* = 0.892, *p* < 0.001, 95% CI = [0.471, 1.315]); multi-dimensional identity labels also significantly affected regional identity (*β* = −0.836, *p* < 0.001, 95% CI = [−1.175, −0.497]); and regional identity significantly influenced purchase intention (*β* = −0.156, *p* = 0.029, 95% CI = [−0.296, −0.015]). Thus, regional identity partially mediated the relationship between multi-dimensional identity labels and purchase intention (*β* = 0.130, SE = 0.065, 95% CI = [0.009, 0.149]), as shown in Figure 3, validating Hypothesis H2.

Control Variable Analysis: To further enhance the accuracy of the experimental results, we included mood as a covariate in a covariance analysis. The results showed that the impact of multi-dimensional identity labels on purchase intention remained significant even after controlling for emotion (*F*(1,282) = 6.206, *p* = 0.013). Thus, emotion did not significantly influence purchase intention, further validating Hypothesis H1.

### 5.3 Interpretation of results

Experiment 2 again confirmed that multi-dimensional identity labels significantly influence consumer purchase intention (F(1,282) = 24.301, *p* < 0.001, η^2^ = 0.079), addressing Hypothesis H1, while regional identity mediated this relationship (indirect effect *β* = 0.130, SE = 0.065, 95% CI = [0.009, 0.149]), supporting Hypothesis H2. Specifically, when consumers strongly identify with local culture, traditions, or regional characteristics, multi-dimensional identity labels evoke emotional resonance, more effectively transforming into purchase behavior. This suggests that the full activation of consumer motivation requires multi-dimensional identity labels to establish an emotional connection through regional identity. These findings extend multiple social categorization theory by demonstrating how intersecting identities (economic-administrative and cultural-territorial) reinforce self-concept alignment, leading to behavioral intentions (52). Additionally, by controlling for mood, we strengthened the robustness of the experiment (effect remains significant, *F*(1,282) = 6.206, *p* = 0.013).

While Experiment 2 explored the relationship between multi-dimensional identity labels and purchase intention from the perspective of individual identity, it did not analyze the role of regional attitudes. Therefore, in Experiment 3, we examined how regional attitudes moderate this relationship.

## 6 Experiment 3: The moderating role of regional attitudes

### 6.1 Experimental design

Experiment 3 adopted a 2 (identity label: multi-dimensional vs. single) × 2 (regional attitude: high vs. low) factorial design to test the moderating role of regional attitudes in the relationship between multi-dimensional identity labels and purchase intention. We recruited 282 participants using the professional data collection platform Credamo, with demographic details provided in Table 1. Participants were randomly assigned to either the single identity group (*N* = 137) or the multi-dimensional identity group (*N* = 145), with the marketing scenario set in a local organic food specialty store.

**Figure 4:**
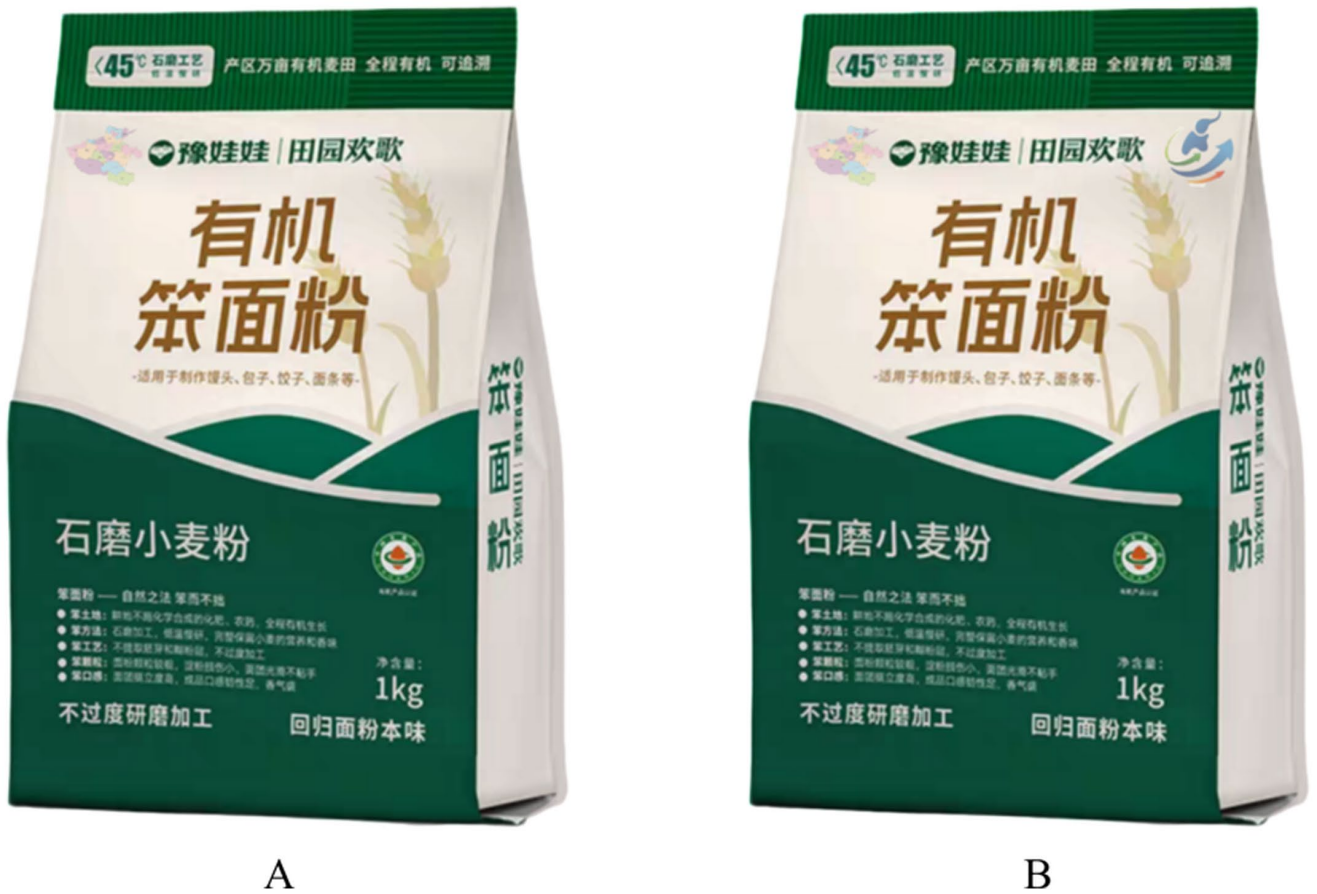
Stimulus materials used in experiment 3 to manipulate identity labels for organic food packaging. Panel **A** shows the single identity label condition using the outline of Henan Province, focusing on provincial identity. Panel **B** shows the multi-dimensional identity label condition, including both the Henan Province outline and the Henan Free Trade Zone label, to assess moderation by regional attitudes.

Experimental Procedure: Participants were instructed to imagine they were selecting appropriate organic food in a renowned local organic food specialty store. They were then provided with an introduction to the organic food used in the experiment, as detailed in Appendix B-3. Participants in the multi-dimensional identity group were shown food advertisements featuring both the Henan Free Trade Zone and a regional outline of Henan; those in the single identity group were shown advertisements featuring only the Henan regional outline, as illustrated in Figure 4. To further validate the manipulation, participants were asked about the label information versatility, for example, “The description of Henan as the production area emphasizes multiple layers of meaning?” Following this, participants responded to questions assessing regional attitudes, such as “I find the production area of the aforementioned food very attractive” (1 = Strongly Disagree, 7 = Strongly Agree) (72)(Cronbach’s *α* = 0.708, χ^2^/df = 1.877, GFI = 0.993, AGFI = 0.967, RMSEA = 0.056, CFI = 0.969, TLI = 0.907). Finally, participants answered questions regarding purchase intention and provided necessary demographic information.

### 6.2 Research results

Manipulation Check: Using label information versatility as a test variable, we conducted an independent samples t-test. The results indicated that organic foods with multi-dimensional identity labels (M = 4.489, SD = 1.814) were perceived as having higher information diversity compared to single identity labels (M = 3.533, SD = 2.011, *t* = 4.199, *p* < 0.001, Cohen d = 1.913), confirming the effectiveness of the manipulation in Experiment 3.

Main Effect Analysis: Using organic food production area identity labels (multi-dimensional vs. single) as the independent variable and purchase intention as the dependent variable, we conducted a single-factor ANOVA. The results showed that consumers exhibited higher purchase intention for multi-dimensional identity labels (M = 6.16, SD = 0.814) compared to single identity labels (M = 5.74, SD = 1.742, *F*(1,280) = 6.896, *p* = 0.009, η^2^ = 0.024), thereby validating Hypothesis H1.

**Figure 5:**
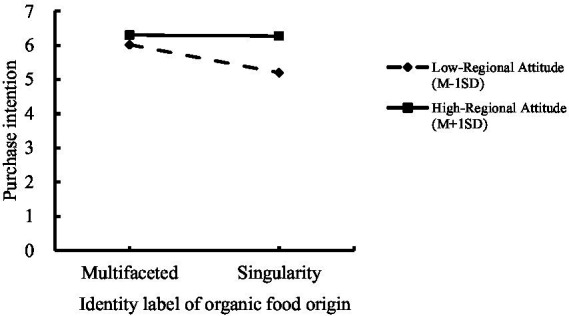
Interaction result diagram depicting the moderating effect of regional attitudes on the relationship between identity labels and purchase intention. The plot illustrates how purchase intention varies across low and high levels of regional attitudes for single vs. multi-dimensional labels (interaction *β* = 0.565, *p* = 0.011, 95% CI = [0.131, 0.998]). Higher regional attitudes amplify the positive effect of multi-dimensional labels, based on Process Model 1 with 5,000 bootstraps.

Moderation Effect Test: We included regional attitude as a moderating variable, with identity label (multi-dimensional vs. single) as the independent variable and purchase intention as the dependent variable, using Process Model 1 to examine the moderating role of regional attitudes (Bootstrap sample: 5000) (78). The results showed that multi-dimensional identity labels significantly influenced purchase intention (*β* = −0.423, *p* = 0.006, 95% CI = [−0.725, −0.121]); multi-dimensional identity labels also significantly affected regional attitudes (*β* = 0.477, *p* < 0.001, 95% CI = [0.259, 0.695]); and the interaction between multi-dimensional identity labels and regional attitudes significantly influenced purchase intention (*β* = 0.565, *p* = 0.011, 95% CI = [0.131, 0.998]). Thus, regional attitudes significantly moderated the relationship between multi-dimensional identity labels and purchase intention, as shown in Figure 5, validating Hypothesis H3.

### 6.3 Interpretation of results

Experiment 3 verified the interaction effect of regional attitudes and multi-dimensional identity labels on purchase intention (interaction *β* = 0.565, *p* = 0.011, 95% CI = [0.131, 0.998]), supporting Hypothesis H3. Specifically, compared to single identity labels, multi-dimensional identity labels more effectively aroused consumer purchase intention (*F*(1,280) = 6.896, *p* = 0.009, η^2^ = 0.024). However, under the influence of regional attitudes, consumers with high regional attitudes showed higher purchase intention for single identity labels compared to the combination of low attitudes and multi-dimensional labels. This moderation underscores the boundary conditions of social identity effects, where positive attitudes amplify category synergy but negative ones may attenuate it, consistent with prior work on attitude-identity interactions (49).

## 7 Robustness tests

In reality, product sales processes involve numerous scenarios. Although the experiments in this study endeavored to encompass a variety of product sales contexts, the range of advertising design elements—particularly those related to audio effects, scenarios, and processes—that could be covered across the three experiments remained limited. Single-paper meta-analysis (SPMA) has emerged in recent years as a widely adopted method for robustness checks among scholars. This approach involves conducting a meta-analysis on multiple studies investigating the same phenomenon within a single paper and is utilized for research synthesis, theory testing, and replicability verification. This method has gained extensive traction among domestic and international scholars, demonstrating significant advantages, particularly in examining the replicability and robustness of findings from individual articles (79). By accounting for potential variations in the operationalization of independent variables or the measurement of dependent variables, the SPMA method safeguards the consistency of behavioral effects, thereby enhancing the reliability and replicability of experimental results (79).

**Figure 6:**
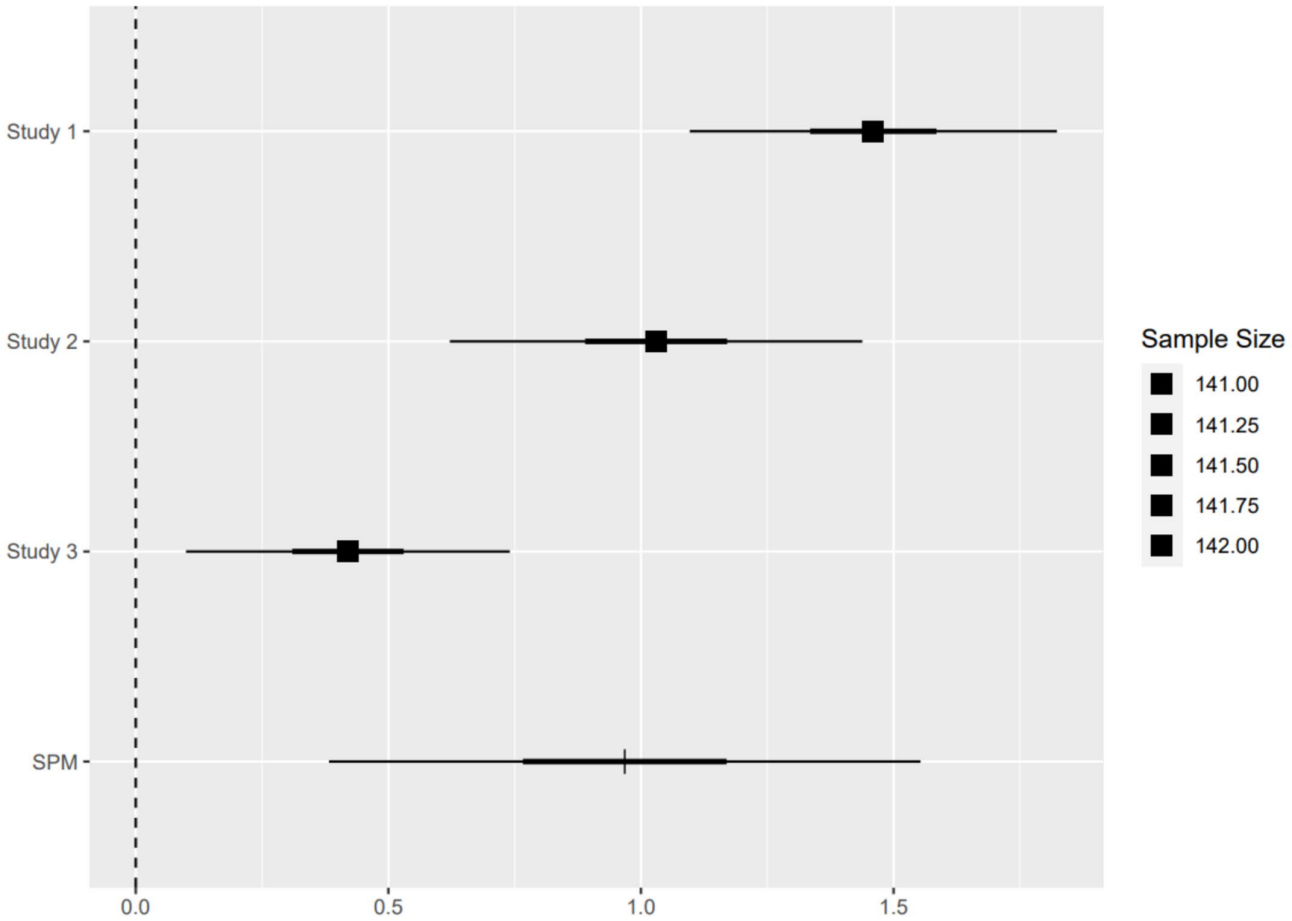
Estimated effects of the four studies.

To mitigate the potential influence of differing operationalizations of the multi-dimensional identity labels and to ensure the reliability and replicability of our findings, this study further employed a single-paper meta-analysis to examine the robustness of the experimental results. As summarized in Table 3, we first detailed the experimental materials and statistics from the three studies. The results of the single-paper meta-analysis revealed a statistically significant difference between the estimated values for the multi-dimensional identity labels and the single identity label (Estimate *β* = 0.968, SE = 0.299, Z = 3.24, *p* = 0.001), as illustrated in Figure 6. This indicates that the results of this study are robust. The conclusions drawn from experiments involving different operationalizations of the process advertisements are reliable, thereby providing further support for the reinforcing effect of multi-dimensional identity labels on organic food.

**Table 3:** Summary of the results of the single-article meta-analysis.

Study	Factor	M	SD	N
Study 1	Multi-dimensional identity labels	5.80	1.456	146
	Single identity labels	4.34	1.643	136
Study 2	Multi-dimensional identity labels	5.72	1.447	144
	Single identity labels	4.69	2.010	140
Study 3	Multi-dimensional identity labels	6.16	0.814	145
	Single identity labels	5.74	1.742	137

## 8 Discussion

Building on the individual interpretations of results from Experiments 1–3, which collectively validate the main effects, mediation, and moderation hypotheses, this section synthesizes the findings to highlight broader theoretical contributions, practical implications, managerial recommendations, and limitations.

### 8.1 Theoretical contributions

First, this study integrates multiple social categorization theory with social identity theory to explain why dual-identity labels outperform single labels. We reveal that Free Trade Zone and provincial labels activate complementary social categories (economic-administrative vs. cultural-territorial), creating a compound regional narrative that enriches consumers’ self-concept. Social Identity Theory suggests that an individual’s self-concept is partly derived from their membership in social groups, and this sense of group belonging significantly influences their behavioral choices (75). In consumer decision-making, consumers tend to choose foods that reinforce their social identity (80). This study, through three scenario-based experiments, validated that multi-dimensional identity labels for organic food production areas, such as those simultaneously indicating both a free trade zone and a provincial administrative region, significantly enhance consumer purchase intention compared to single identity labels. This finding not only confirms the applicability of Social Identity Theory in explaining consumer behavior but also reveals the synergistic effects of multiple identities. The results demonstrate that multi-dimensional identity labels, by activating consumers’ regional identity, strengthen their connection to the food, particularly when consumers exhibit strong regional identification (81). This extends the application of Social Identity Theory, showcasing its potential in shaping consumer preferences through labeling strategies in a globalized context. Furthermore, the study highlights the role of identity salience in consumer contexts, where the activation of multiple identities leads to behaviors consistent with those identities. This mechanism provides new theoretical insights into how social identity influences behavior in the context of region-specific foods like organic products, deepening our understanding of identity-driven consumer behavior.

Moreover, this study makes significant contributions to the understanding of the relationship between food labeling and consumer perceptions. Food labels, as a critical tool for communicating food attributes and origins, play a central role in shaping consumer perceptions and purchasing decisions (82). Previous research has primarily focused on the impact of single-attribute labels, such as nutritional or geographical labels, on consumer behavior (83). However, this study is the first to systematically explore the unique effects of multi-dimensional identity labels. The experimental results indicate that multi-dimensional identity labels, by providing a richer narrative about food, significantly enhance consumer perceptions of organic food quality, safety, and environmental sustainability, thereby increasing purchase intention. These findings offer new empirical support for brand positioning theory (84), demonstrating that brands can establish a competitive advantage by integrating multiple identity labels in a market characterized by intense competition. Additionally, this research reveals the critical role of emotional connections in consumer perceptions, with multi-dimensional identity labels strengthening the emotional bond between consumers and food by evoking regional identity. This not only enriches the theoretical framework of emotional marketing but also provides theoretical support for leveraging label design to enhance consumer trust and preference. Compared to single labels, the narrative complexity of multi-dimensional identity labels opens new possibilities for marketing strategies, particularly in the organic food sector, where authenticity and traceability are paramount.

Finally, this study deepens the understanding of identity recognition mechanisms in consumer behavior motivation. By introducing regional identity as a mediating variable, the study clarifies how multi-dimensional identity labels influence purchase intention by activating consumers’ social identity. According to Identity Theory (85), individuals’ behavioral choices are driven by their identity recognition, and when a specific identity is activated, they are more likely to engage in behaviors consistent with that identity. This study found that multi-dimensional identity labels are more effective in arousing consumers’ regional identity, subsequently translating into purchase intention for organic food. The validation of this mediating effect not only provides new evidence for the motivational mechanisms in consumer behavior research but also highlights the central role of identity recognition in label strategies. Furthermore, by controlling for variables such as mood, the study excluded other potential confounding factors, further strengthening the robustness of its conclusions. This discovery provides theoretical support for market segmentation in marketing theory, indicating that multi-dimensional identity labels can more precisely evoke purchase motivation in consumer groups with strong regional identification, enriching our understanding of the causal chain in consumer behavior.

### 8.2 Practical implications

In addition to its theoretical contributions, this study offers significant practical implications. First, the findings reveal the substantial impact of multi-dimensional identity labels on consumer purchase intention for organic food, providing important practical guidance for marketers. The results demonstrate that compared to single identity labels, multi-dimensional identity labels, such as those indicating both a free trade zone and a provincial administrative region, significantly enhance consumer purchase intention. This effect is strengthened through the mediating role of regional identity and moderated by regional attitudes. For organic food marketers operating in free trade zones, this finding suggests that highlighting multi-dimensional identity labels on food packaging and promotional materials can optimize branding strategies. For example, organic food sold in the Hainan Free Trade Zone can emphasize both “Hainan Free Trade Zone” and “Hainan Province,” combining the modern, economic image of the free trade zone with Hainan’s tropical ecological culture to enhance the food’s appeal and credibility. This dual labeling not only enhances the authenticity and traceability of the food but also meets consumers’ demands for transparency in organic food production. To implement this strategy, marketers should conduct market research (e.g., surveys or focus groups) to deeply understand the regional identity and attitudes of their target market, tailoring label designs and promotional content accordingly. For instance, if target consumers hold positive attitudes toward both the high-quality regulation of free trade zones and the cultural traditions of the province, marketers can emphasize these qualities in advertisements to strengthen emotional connections. Additionally, marketers must ensure consistency in label information across packaging, online ads, and social media to build a unique value proposition based on regional characteristics and free trade zone economic advantages. This strategy not only helps increase consumer purchase intention and brand loyalty but also provides a practical pathway for organic food to stand out in a competitive market.

The study’s conclusions also provide practical evidence for policymakers aiming to support the development of local organic food industries. The results demonstrate that multi-dimensional identity labels significantly enhance purchase intention by strengthening consumers’ regional identity and trust, offering theoretical support for designing corresponding policy measures. Policymakers can implement economic incentive policies to encourage organic food producers to adopt multi-dimensional identity labels. For example, they can offer tax exemptions or marketing subsidies to enterprises that meet dual labeling standards, promoting a unified brand image that combines free trade zone and provincial identities. Policymakers can also collaborate with industry associations and marketing experts to develop standardized labeling guidelines, ensuring the authenticity and consistency of labels. For instance, they can establish a certification system similar to France’s “appellation d’origine contrôlée” to verify the legitimacy of multi-dimensional identity labels, thereby boosting consumer confidence in food quality. Additionally, policymakers can invest in public awareness campaigns through television, social media, and community events to educate consumers about the benefits of organic food and the significance of regional labels. For example, in the Henan Free Trade Zone, promotional activities can highlight its dual advantages as an agricultural powerhouse and a free trade zone to deepen consumers’ sense of identification with local organic food. Furthermore, policymakers can collaborate with educational institutions to incorporate knowledge about regional organic food production into school curricula, fostering long-term consumer awareness. Through these measures, policymakers can not only stimulate market demand for organic food but also enhance regional economic vitality and the reputation of sustainable agriculture. Aligning policy initiatives with consumer behavior provides strategic support for free trade zones to achieve broader economic objectives.

### 8.3 Managerial recommendations

Designers should visually integrate multi-dimensional identity cues into packaging through clear hierarchy and balance. The economic-administrative attribute (e.g., Free Trade Zone label) ought to be represented with clean, modern typographic elements or minimalistic symbols to convey professionalism and regulatory superiority. In contrast, the cultural-territorial element (e.g., provincial outline or local heritage symbol) should use softer visual motifs, color gradients, or traditional cultural imagery. Combining these layers strategically enhances informational richness while maintaining simplicity, avoiding the “visual overload” risk.

Retailers can segment customers by regional attitudes. In regions with strong local pride, promotional materials should emphasize the provincial identity to resonate emotionally. Conversely, in cosmopolitan or globally oriented markets, highlighting the Free Trade Zone brand image as a mark of quality and innovation will better align with consumers’ aspirations. Retailers may also employ location-based digital marketing to tailor advertisements dynamically according to consumers’ regional affiliations.

Regulatory agencies should consider developing dual certification frameworks that verify both administrative and cultural origin authenticity. This can involve a combined audit system ensuring traceability and consumer transparency, similar to EU’s PDO/PGI labeling synergy. Early implementation of such cross-label standards will improve export competitiveness and reinforce consumer confidence in certified organic products.

In an increasingly globalized food economy, this dual-identity approach offers a branding model flexible enough to balance global trustworthiness with local embeddedness. International brands could leverage multi-layered origin storytelling, pairing sustainability claims with culturally resonant geographies, thus fostering identity-based differentiation in saturated markets.

### 8.4 Limitations and directions for future research

One notable limitation of this study is its sample selection and geographic scope, which may limit the generalizability of its findings. The study was conducted exclusively in China, focusing on free trade zones and provincial administrative regions in Xinjiang Uyghur Autonomous Region, Hainan Province, and Henan Province, with a total of 848 participants across three experiments. While these regions provide valuable insights into China’s organic food market, the results may not fully apply to consumers in other countries or cultures with differing views on free trade zones or regional identity. For example, in markets where free trade zones are less developed or regional identity has a weaker influence on food perceptions (e.g., certain regions in North America or Europe), the impact of multi-dimensional identity labels on purchase intention may differ due to variations in economic structures, cultural values, or consumer priorities. Additionally, the study’s focus on urban consumers in China may overlook rural or socioeconomically diverse populations, further limiting its scope. To address cultural generalizability, future research should test these effects in cross-cultural contexts, such as comparing Chinese consumers with those in the EU (where geographical indications like Protected Designation of Origin are prominent) or the US (with varying state-level identities). Empirical evidence suggests cultural background significantly influences label perception and food acceptability, with Eastern cultures potentially prioritizing collective regional identities more than individualistic Western ones (86–88). This could involve multi-country experiments to validate the model’s robustness across diverse cultural settings. Incorporating a broader range of consumer demographics, including rural populations and varied socioeconomic groups, will also enhance the robustness of the results. These efforts will provide a more comprehensive understanding of how multi-dimensional identity labels influence organic food purchase intention globally, offering actionable insights for international marketing strategies.

The experimental design, while methodologically sound, has certain limitations worth considering. The study employed online scenario-based experiments to simulate consumer decisions, involving hypothetical purchasing scenarios in settings such as shopping malls or specialty stores. While this approach allows for controlled testing of label effects, it may not fully replicate the complexity of real-world purchasing environments, where factors such as price fluctuations, in-store promotions, or sensory experiences (e.g., food smell, texture) may influence decisions. For example, the online format may fail to capture impulse purchases or social dynamics during shopping, potentially reducing the ecological validity of the findings. Furthermore, the reliance on self-reported measures of purchase intention introduces the risk of response bias, such as social desirability, where participants may exaggerate their willingness to buy organic food to align with perceived social norms.

To strengthen the justification for the external validity of these online experiments, it is important to note that scenario-based simulations have been widely validated in consumer behavior research for their ability to approximate real decision-making processes while maintaining high internal control (89, 90). Specifically, empirical studies demonstrate that online experiments can yield reliable and generalizable insights into sustainable consumption behaviors (91), with ecological validity enhanced through realistic stimuli and diverse participant recruitment. Moreover, research on contextual methodologies in sensory and consumer studies supports the use of such simulations to balance experimental rigor with practical relevance, showing comparable outcomes to field observations in labeling effects (92). Nonetheless, this limitation highlights the need for caution in extrapolating results to actual market behaviors.

Additionally, a practical risk not fully explored is over-labeling, where excessive multi-dimensional labels could lead to information overload, consumer confusion, or skepticism. Research on front- and back-of-pack labeling indicates that too many claims can overwhelm cognitive processing, reducing trust and purchase intention rather than enhancing it (93). For instance, cluttered packaging might be perceived as manipulative marketing, eroding authenticity in organic food contexts. Future research should conduct field experiments in real-world retail environments (e.g., organic food markets or supermarkets) to observe actual consumer behavior, addressing these limitations. Supplementing these methods with advanced technologies, such as eye-tracking for label attention or point-of-sale data for actual purchases, can provide more objective insights into how multi-dimensional identity labels influence decision-making. By bridging the gap between controlled experiments and real-world contexts, such studies will enhance the practical applicability of the findings for organic food marketers.

Another limitation is that our experimental design adopted a price-controlled scenario, which excludes potential interactions between price sensitivity and label perception. While this decision allows for a cleaner test of psychological mechanisms, real-world purchase decisions often involve trade-offs between price and authenticity. Future research should integrate price-level manipulations (e.g., high vs. low pricing) to examine whether the premium consumers are willing to pay for multi-dimensional identity-labeled organic food differs across income and education groups. This approach will enhance external validity and deepen understanding of how socio-economic contexts shape label effectiveness.

Finally, this study may have limitations in variable measurement and control, potentially affecting the depth of its conclusions. Although the study effectively demonstrated the mediating and moderating roles of regional identity and attitudes in the relationship between multi-dimensional identity labels and purchase intention, it did not comprehensively explore other potential influencing factors. Consumer characteristics such as age, income, educational level, or prior knowledge of organic food were not systematically examined as moderating factors. Similarly, external variables such as brand familiarity, food pricing, or competitive label claims were excluded, despite their potential interactions with identity labels in real-world purchasing scenarios. The study’s reliance on self-reporting scales also raises concerns about measurement reliability, as these scales may be subject to participants’ subjective biases or inconsistent interpretations. To overcome these limitations, future research should adopt a more comprehensive approach by including a broader range of consumer and contextual variables in the analysis. Employing multi-method measurement techniques, such as combining survey data with behavioral experiments or physiological tools, will enhance the accuracy of the results. Additionally, investigating the interactions between multiple label types and other certifications can reveal synergistic or competitive effects, providing more detailed insights into label design strategies. This will deepen the theoretical and practical understanding of how identity-based labels drive organic food consumption.

## 9 Conclusion

This study demonstrates that multi-dimensional identity labels, integrating free trade zone and provincial administrative region identifiers, significantly enhance consumer purchase intention for organic food through the mediating role of regional identity and moderated by regional attitudes. These insights advance social identity and multiple social categorization theories in the domain of food labeling and provide practical strategies for marketers and policymakers in promoting sustainable organic consumption. However, as the findings are context-specific to Chinese free trade zones, shaped by unique economic policies and cultural emphases on regional affiliation, caution is warranted in generalizing to other global contexts. Future research should extend these investigations to diverse international settings to assess the universality of multi-dimensional labeling effects.

## Data Availability

The raw data supporting the conclusions of this article will be made available by the authors, without undue reservation.

## Footnotes

1
https://www.credamo.com

## References

[1] Moroșan E Popovici V Popescu IA Daraban A Karampelas O Matac LM . Perception, trust, and motivation in consumer behavior for organic food acquisition: an exploratory study. Foods. (2025) 14:293. doi: 10.3390/foods14020293, PMID: 39856959 PMC11765215

[2] Abbasi E. Edible insects as a sustainable and innovative approach to addressing global food security and environmental challenges: a comprehensive review. J. Insects Food Feed. (2025) 1:1–12.

[3] Nirmal N Anyimadu CF Khanashyam AC Bekhit AEA Dhar BK. Alternative protein sources: addressing global food security and environmental sustainability. Sustain. Dev. (2025) 33:3958–69. doi: 10.1002/sd.3338

[4] Ikram A Mehmood H Arshad MT Rasheed A Noreen S Gnedeka KT. Applications of artificial intelligence (AI) in managing food quality and ensuring global food security. CyTA-J Food. (2024) 22:2393287. doi: 10.1080/19476337.2024.2393287

[5] Liu H Meng J Bo W Cheng D Li Y Guo L. Biodiversity management of organic farming enhances agricultural sustainability. Sci. Rep. (2016) 6:23816. doi: 10.1038/srep23816, PMID: 27032369 PMC4817119

[6] Barański M Średnicka-Tober D Volakakis N Seal C Sanderson R Stewart GB. Higher antioxidant and lower cadmium concentrations and lower incidence of pesticide residues in organically grown crops: a systematic literature review and meta-analyses. Br. J. Nutr. (2014) 112:794–811. doi: 10.1017/S0007114514001366, PMID: 24968103 PMC4141693

[7] Hurtado-Barroso S Tresserra-Rimbau A Vallverdú-Queralt A Lamuela-Raventós RM. Organic food and the impact on human health. Crit. Rev. Food Sci. Nutr. (2019) 59:704–14. doi: 10.1080/10408398.2017.1394815, PMID: 29190113

[8] Kuang T Yang D Zou D. The impact of transparent packaging: how transparent packaging for organic foods affects tourists' green purchasing behavior. Front Nutr. (2024) 11:1328596. doi: 10.3389/fnut.2024.1328596, PMID: 38406189 PMC10885356

[9] Galutskykh N Didorchuk I. The current trends in the world market of organic products. Business Inform. (2024) 2:20–6. doi: 10.32983/2222-4459-2024-2-20-26

[10] Aschemann-Witzel J Zielke S. Can't buy me green? A review of consumer perceptions of and behavior toward the price of organic food. J. Consum. Aff. (2017) 51:211–51. doi: 10.1111/joca.12092

[11] Melović B Ćirović D Dudić B Vulic T Greguš M. The analysis of marketing factors influencing consumers’ preferences and acceptance of organic food products—recommendations for the optimization of the offer in a developing market. Foods. (2020) 9:259. doi: 10.3390/foods9030259 32121318 PMC7142824

[12] Cao Y Miao L. Consumer perception of clean food labels. Br. Food J. (2022) 125:433–48. doi: 10.1108/BFJ-03-2021-0246

[13] Silva B Lima J Baltazar A Pinto E Fialho S. Perception of Portuguese consumers regarding food labeling. Nutrients. (2022) 14:2944. doi: 10.3390/nu14142944, PMID: 35889901 PMC9323138

[14] Wang L Yang D. How recycling and transformation information influences tourists’ purchase intentions towards green food products. CyTA-J Food. (2025) 23:2528540. doi: 10.1080/19476337.2025.2528540

[15] Aitken R Watkins L Williams J Kean A. The positive role of labelling on consumers’ perceived behavioural control and intention to purchase organic food. J. Clean. Prod. (2020) 255:120334. doi: 10.1016/j.jclepro.2020.120334

[16] Liang A Lim W. Why do consumers buy organic food? Results from an S–O–R model. Asia Pac. J. Mark. Logist. (2020) 33:394–415. doi: 10.1108/APJML-03-2019-0171

[17] Yang D Gui G Yao Y Ke X. Effect on consumers’ sustainable purchase intention of dietary supplement purine labeling. Front Nutr. (2025) 12:1526713. doi: 10.3389/fnut.2025.1526713, PMID: 40535036 PMC12173902

[18] Santeramo FG Lamonaca E. Evaluation of geographical label in consumers’ decision-making process: a systematic review and meta-analysis. Food Res. Int. (2020) 131:108995. doi: 10.1016/j.foodres.2020.108995 32247446

[19] Aprile MC Caputo V Nayga JRM. Consumers' valuation of food quality labels: the case of the European geographic indication and organic farming labels. Int. J. Consum. Stud. (2012) 36:158–65. doi: 10.1111/j.1470-6431.2011.01092.x

[20] Glogovețan A-I Pocol CB. The role of promoting agricultural and food products certified with European union quality schemes. Foods. (2024) 13:970. doi: 10.3390/foods13060970, PMID: 38540960 PMC10969692

[21] Chen N-H. Geographical indication labelling of food and behavioural intentions. Br. Food J. (2021) 123:4097–115. doi: 10.1108/BFJ-06-2020-0552

[22] Milano MZ Cazella AA. Environmental effects of geographical indications and their influential factors: a review of the empirical evidence. Current Res Environ Sustain. (2021) 3:100096. doi: 10.1016/j.crsust.2021.100096

[23] Maldonado L Farias S Da Cruz KV Santos BPD Castro L Castro I. Marketing communication strategies on labels of food products consumed by children. Rev. Saude Publica. (2023) 57:92. doi: 10.11606/s1518-8787.2023057004614

[24] Alsem K Kostelijk E. Identity based marketing: a new balanced marketing paradigm. Eur. J. Mark. (2008) 42:907–14. doi: 10.1108/03090560810891064

[25] Nagy LB Lakner Z Temesi Á. Is it really organic? Credibility factors of organic food–a systematic review and bibliometric analysis. PLoS One. (2022) 17:e0266855. doi: 10.1371/journal.pone.0266855, PMID: 35421157 PMC9009713

[26] Watanabe E Alfinito S Barbirato LL. Certification label and fresh organic produce category in an emerging country: an experimental study on consumer trust and purchase intention. Br. Food J. (2021) 123:2258–71. doi: 10.1108/BFJ-09-2020-0808

[27] Bao T Dai Y Feng Y Liu S Wang R. Trade liberalization and trade and capital flows: evidence from China pilot free trade zones 1. World Econ. (2023) 46:1408–22. doi: 10.1111/twec.13387

[28] Chen H Yuan B Cui Q. Does the pilot free trade zone policy attract the entering of foreign-invested enterprises? The evidence from China. Appl. Econ. Lett. (2020) 28:1162–8. doi: 10.1080/13504851.2020.1803482

[29] Zhao Y Wang S Liu X Tang X. Effect of the logistics industry on the promotion of China's position in the global value chain: an international trade perspective. Int. Rev. Econ. Financ. (2023) 86:834–47. doi: 10.1016/j.iref.2023.03.029

[30] Weinrich R Spiller A. Developing food labelling strategies: multi-level labelling. J. Clean. Prod. (2016) 137:1138–48. doi: 10.1016/j.jclepro.2016.07.156

[31] Akter S Ali S Fekete-Farkas M Fogarassy C Lakner Z. Why organic food? Factors influence the organic food purchase intension in an emerging country (study from northern part of Bangladesh). Resources. (2023) 12:5. doi: 10.3390/resources12010005

[32] Hornsey MJ. Social identity theory and self-categorization theory: a historical review. Soc. Personal. Psychol. Compass. (2008) 2:204–22. doi: 10.1111/j.1751-9004.2007.00066.x

[33] Hogg MA Abrams D Brewer MB. Social identity: the role of self in group processes and intergroup relations. Group Process Intergroup Relat. (2017) 20:570–81. doi: 10.1177/1368430217690909

[34] Ellemers N Spears R Doosje B. Self and social identity. Annu. Rev. Psychol. (2002) 53:161–86. doi: 10.1146/annurev.psych.53.100901.135228 11752483

[35] Abbink K Harris D. In-group favouritism and out-group discrimination in naturally occurring groups. PLoS One. (2019) 14:e0221616. doi: 10.1371/journal.pone.0221616, PMID: 31483822 PMC6726232

[36] Aboud FE. The formation of in-group favoritism and out-group prejudice in young children: are they distinct attitudes? Dev. Psychol. (2003) 39:48–60. doi: 10.1037/0012-1649.39.1.48, PMID: 12518808

[37] Park LE Maner JK. Does self-threat promote social connection? The role of self-esteem and contingencies of self-worth. J. Pers. Soc. Psychol. (2009) 96:203–17. doi: 10.1037/a0013933, PMID: 19210075

[38] Panzone L Di Vita G Borla S D’Amico M. When consumers and products come from the same place: preferences and WTP for geographical indication differ across regional identity groups. J. Int. Food Agribus. Mark. (2016) 28:286–313. doi: 10.1080/08974438.2016.1145611

[39] Chattaraman V Lennon SJ Rudd NA. Social identity salience: effects on identity-based brand choices of Hispanic consumers. Psychol. Mark. (2010) 27:263–84. doi: 10.1002/mar.20331

[40] Sidorenkov AV Borokhovski EF. The role of cohesion and productivity norms in performance and social effectiveness of work groups and informal subgroups. Behav. Sci. (2023) 13:361. doi: 10.3390/bs13050361, PMID: 37232598 PMC10215452

[41] Forsyth DR. Recent advances in the study of group cohesion. Group Dyn. Theory Res. Pract. (2021) 25:213–28. doi: 10.1037/gdn0000163

[42] Hill NS Villamor I. The influence of team cultural value orientations on norms of conduct in hybrid teams: implications for team cohesion and performance. Group Process Intergroup Relat. (2023) 26:1436–56. doi: 10.1177/13684302221123922

[43] Carrión Bósquez NG Arias-Bolzmann LG Martinez Quiroz AK. The influence of price and availability on university millennials’ organic food product purchase intention. Br. Food J. (2023) 125:536–50. doi: 10.1108/BFJ-12-2021-1340

[44] Arshad M Qasim N Farooq O Rice J. Empowering leadership and employees' work engagement: a social identity theory perspective. Manag. Decis. (2022) 60:1218–36. doi: 10.1108/MD-11-2020-1485

[45] Reinders JJ Krijnen W. Interprofessional identity and motivation towards interprofessional collaboration. Med. Educ. (2023) 57:1068–78. doi: 10.1111/medu.15096, PMID: 37073763

[46] Oakes T. China's provincial identities: reviving regionalism and reinventing “Chineseness”. J. Asian Stud. (2000) 59:667–92.

[47] Lei T Xie P. Fostering enterprise innovation: the impact of China’s pilot free trade zones. J. Knowl. Econ. (2024) 15:10412–41. doi: 10.1007/s13132-023-01501-8

[48] Haratbar HR Popkowski Leszczyc PT Gonzalez-Arcos C. The effect of self-concept components on sustainable food choice. Bus. Strateg. Environ. (2025) 34:8667–85. doi: 10.1002/bse.70034

[49] Steenkamp J De Jong M. A global investigation into the constellation of consumer attitudes toward global and local products. J. Mark. (2010) 74:18–40. doi: 10.1509/jmkg.74.6.18

[50] Poeggel K. You are where you eat: a theoretical perspective on why identity matters in local food groups. Front Sustain Food Syst. (2022) 6:782556. doi: 10.3389/fsufs.2022.782556

[51] Zaharia A Gonța I. The healthy eating movement on social media and its psychological effects on body image. Front Nutr. (2024) 11:1474729. doi: 10.3389/fnut.2024.1474729, PMID: 39742097 PMC11685096

[52] Roccas S Brewer MB. Social identity complexity. Personal. Soc. Psychol. Rev. (2002) 6:88–106. doi: 10.1207/S15327957PSPR0602_01

[53] Crisp RJ Hewstone M. Multiple social categorization. Adv. Exp. Soc. Psychol. (2007) 39:163–254. doi: 10.1016/S0065-2601(06)39004-1

[54] Dong L Tian K. The use of Western brands in asserting Chinese national identity. J. Consum. Res. (2009) 36:504–23. doi: 10.1086/598970

[55] Muzzioli L Maddaloni L Pintavalle M Poggiogalle E Di Vincenzo O Migliaccio S . Toward multidimensional front-of-pack labels: integrating nutritional, environmental, and processing information. Nutrients. (2025) 17:2258. doi: 10.3390/nu17142258, PMID: 40732883 PMC12298846

[56] Sá A Carmo LM Valencia G. The influence of clean food labels on consumers' perception. Packag. Technol. Sci. (2023) 36:757–66. doi: 10.1002/pts.2757

[57] Jensen J Christensen T Denver S Ditlevsen K Lassen J Teuber R. Heterogeneity in consumers' perceptions and demand for local (organic) food products. Food Qual. Prefer. (2019) 73:255–65. doi: 10.1016/j.foodqual.2018.11.002

[58] Pedersen S Aschemann-Witzel J Thøgersen J. Consumers' evaluation of imported organic food products: the role of geographical distance. Appetite. (2017) 130:134–45. doi: 10.1016/j.appet.2018.08.016 30114490

[59] Bi S Shao L Tu C-H Lai W Cao Y Hu J. Achieving carbon neutrality: the effect of China pilot free trade zone policy on green technology innovation. Environ. Sci. Pollut. Res. (2023) 30:50234–47. doi: 10.1007/s11356-023-25803-1, PMID: 36790713

[60] Xu S Shen R Zhang Y Cai Y. Fostering regional innovation efficiency through pilot free trade zones: evidence from China. Econ Anal Policy. (2023) 81:356–67. doi: 10.1016/j.eap.2023.12.004

[61] Cao Z Mustafa M Isa MHM. Regional architecture building identity: the mediating role of authentic pride. Buildings. (2024) 14:1059. doi: 10.3390/buildings14041059

[62] Thøgersen J. How does origin labelling on food packaging influence consumer product evaluation and choices? A systematic literature review. Food Policy. (2023) 119:102503. doi: 10.1016/j.foodpol.2023.102503

[63] Dorisse A Charry K Parguel B. When multi-labelling backfires: the influence of sustainability FOP labels valence and orientation. Appetite. (2025) 213:108055. doi: 10.1016/j.appet.2025.108055, PMID: 40354982

[64] Raagmaa G. Regional identity in regional development and planning1. Eur. Plan. Stud. (2002) 10:55–76. doi: 10.1080/09654310120099263

[65] Scarpa R Philippidis G Spalatro F. Product-country images and preference heterogeneity for Mediterranean food products: a discrete choice framework. Agribusiness. (2005) 21:329–49. doi: 10.1002/agr.20051

[66] Kuznesof S Tregear A Moxey A. Regional foods: a consumer perspective. Br. Food J. (1997) 99:199–206. doi: 10.1108/00070709710181531

[67] Fan M Weiming C. A study of regional image of agricultural product impact on purchase intention. J Soc Sci Stud. (2023) 10:248. doi: 10.5296/jsss.v10i1.21028

[68] He J Wang C. Cultural identity and consumer ethnocentrism impacts on preference and purchase of domestic versus import brands: an empirical study in China. J. Bus. Res. (2015) 68:1225–33. doi: 10.1016/j.jbusres.2014.11.017

[69] Shangguan S Afshin A Shulkin M Ma W Marsden D Smith J. A meta-analysis of food labeling effects on consumer diet behaviors and industry practices. Am. J. Prev. Med. (2019) 56:300–14. doi: 10.1016/j.amepre.2018.09.024, PMID: 30573335 PMC6340779

[70] Bryła P. Regional ethnocentrism on the food market as a pattern of sustainable consumption. Sustainability. (2019) 11:6408. doi: 10.3390/su11226408

[71] Aprile M Caputo V Nayga R. Consumers’ preferences and attitudes toward local food products. J. Food Prod. Mark. (2016) 22:19–42. doi: 10.1080/10454446.2014.949990

[72] Charton-Vachet F Lombart C Louis D. Impact of attitude towards a region on purchase intention of regional products: the mediating effects of perceived value and preference. Int. J. Retail Distrib. Manag. (2020) 48:707–25. doi: 10.1108/IJRDM-09-2019-0315

[73] Van Ittersum K Meulenberg M Trijp H Candel M. Consumers' appreciation of regional certification labels: a pan-European study. J Agric Econ. (2007) 58:1–23. doi: 10.1111/j.1477-9552.2007.00080.x

[74] Wang J Tao J Chu M. Behind the label: Chinese consumers’ trust in food certification and the effect of perceived quality on purchase intention. Food Control. (2020) 108:106825. doi: 10.1016/j.foodcont.2019.106825

[75] Turner JC Brown RJ Tajfel H. Social comparison and group interest in ingroup favouritism. Eur. J. Soc. Psychol. (1979) 9:187–204.

[76] Asún R Zúñiga C Morales J-F. Design and validation of the revised regional identity scale (RIS-2)/Diseño y validación de la Escala de Identidad regional revisada (RIS-2). Int. J. Soc. Psychol. (2018) 33:357–89. doi: 10.1080/02134748.2018.1439691, PMID: 41215551

[77] Liang B Yang D Tan F Sun D Li J. How psychological ownership over nutritional products affects purchase intentions of high-pressure working groups. Front Nutr. (2024) 11:1401035. doi: 10.3389/fnut.2024.1401035, PMID: 39176026 PMC11338884

[78] Igartua J-J Hayes AF. Mediation, moderation, and conditional process analysis: concepts, computations, and some common confusions. Span J Psychol. (2021) 24:e49. doi: 10.1017/SJP.2021.46, PMID: 35923144

[79] McShane BB Böckenholt U. Single-paper meta-analysis: benefits for study summary, theory testing, and replicability. J. Consum. Res. (2017) 43:1048–63. doi: 10.1093/jcr/ucw085

[80] Escalas JE Bettman JR. Self-construal, reference groups, and brand meaning. J. Consum. Res. (2005) 32:378–89. doi: 10.1086/497549

[81] Reed A. Activating the self-importance of consumer selves: exploring identity salience effects on judgments. J. Consum. Res. (2004) 31:286–95. doi: 10.1086/422108

[82] Verbeke W Ward RW. Consumer interest in information cues denoting quality, traceability and origin: an application of ordered probit models to beef labels. Food Qual. Prefer. (2006) 17:453–67. doi: 10.1016/j.foodqual.2005.05.010

[83] Drichoutis AC Lazaridis P Nayga JRM. Consumers' use of nutritional labels: a review of research studies and issues. Acad. Mark. Sci. Rev. (2006) 2006:1

[84] Keller KL. Brand synthesis: the multidimensionality of brand knowledge. J. Consum. Res. (2003) 29:595–600. doi: 10.1086/346254

[85] Burke PJ Stets JE. Identity theory: Revised and expanded. Britain: Oxford University Press (2022).

[86] Jeong S Lee J. Effects of cultural background on consumer perception and acceptability of foods and drinks: a review of latest cross-cultural studies. Curr. Opin. Food Sci. (2021) 42:248–56. doi: 10.1016/j.cofs.2021.07.004

[87] Priya K Babu K. Discovering consumer behavior towards back-of-pack nutrition labels: a systematic literature review. Current Res Nutrition Food Sci J. (2024) 12:502–26. doi: 10.12944/CRNFSJ.12.2.3

[88] Ho KFX Liu F Tarabashkina L Volery T. Cross-cultural differences in consumers' attention to food labels. Br. Food J. (2022) 124:4888–904. doi: 10.1108/BFJ-07-2021-0751

[89] Arias Puentes CP Trujillo CA. The role of online experiments in the understanding of sustainable consumption behaviors: a systematic methodological literature review. Int. J. Consum. Stud. (2025) 49:e70033. doi: 10.1111/ijcs.70033

[90] Plaza AG Delarue J Saulais L. The pursuit of ecological validity through contextual methodologies. Food Qual. Prefer. (2019) 73:226–47. doi: 10.1016/j.foodqual.2018.11.004

[91] Demarque C Charalambides L Hilton DJ Waroquier L. Nudging sustainable consumption: the use of descriptive norms to promote a minority behavior in a realistic online shopping environment. J. Environ. Psychol. (2015) 43:166–74. doi: 10.1016/j.jenvp.2015.06.008

[92] Taufik D Kunz MC Onwezen MC. Changing consumer behaviour in virtual reality: a systematic literature review. Comput Hum Behav Rep. (2021) 3:100093. doi: 10.1016/j.chbr.2021.100093

[93] Roberto CA Ng SW Ganderats-Fuentes M Hammond D Barquera S Jauregui A. The influence of front-of-package nutrition labeling on consumer behavior and product reformulation. Annu. Rev. Nutr. (2021) 41:529–50. doi: 10.1146/annurev-nutr-111120-094932, PMID: 34339293

