# Supplementary material for: Economic empowerment of free trade zone identity labels: the impact of multi-dimensional identity labels on consumer purchase intention for organic food
Source: Front Nutr. 2026 Jan 8;12:1681453. doi: 10.3389/fnut.2025.1681453 (PMC12825458; doi:10.3389/fnut.2025.1681453)
Supplement: Supplementary file 1 [file Table_1.DOCX]

Appendix A:

Table 1. Summary of the measurement items of the Three experiments.

| Variant | Measurement Items | Scale source |
| --- | --- | --- |
| Demographic Information | What is your gender? |  |
|  | What is your age? |  |
|  | What is your education? |  |
| Label Information Complexity | The information about the organic food's origin displayed on the packaging gives you the impression of encompassing multiple different aspects or attributes? |  |
| Label Information Diversity | The description of the food's origin from Hainan Province makes me feel that it emphasizes the multiple identities or characteristics of the origin? |  |
| Label Information Versatility | The description of Henan Province as the origin in the food information presents multiple layers of meaning? |  |
| Emotion | Do you agree that your current mood is positive (happy, pleasant, relaxed)? | (1) |
| Regional Identity | Being a member of this region makes me feel very proud. | (2) |
|  | I feel like I am a part of this region. |  |
|  | I want to live in another region in the near future. |  |
|  | If I have to leave this region for any reason, I will try to come back one day. |  |
|  | If I am away from this region for a long time, I will start to miss the scenery and climate there, as if this region is a part of me. |  |
|  | If someone says something unpleasant about the region's landscape in a conversation with family, friends, or acquaintances, I will probably feel uneasy. |  |
|  | I feel an important connection with the landscape of this region. |  |
|  | I think this region is the most beautiful region in this country. |  |
|  | I feel like I am part of the history of this region. |  |
|  | I am honored to inherit the history of this region, including all its good and bad, which is my region and culture. |  |
|  | To some extent, I reflect the culture of this region. |  |
|  | I am proud to live in such a region with folk tales, customs and traditions. |  |
|  | I feel a certain connection with the people of this region. |  |
|  | When someone praises the residents of my region, I think it is a compliment to me personally. |  |
|  | When I talk about the people of this region, I often say "we" instead of "they". |  |
|  | If someone describes me as a typical person from this region, I will feel good. |  |
| Regional attitude | I think the production place of the aforementioned food is very pleasant. | (3) |
|  | I think the production place of the aforementioned food is very attractive. |  |
|  | I like the production place of the aforementioned food. |  |
|  | Overall, you have a positive attitude towards the production place of the aforementioned food. |  |
| Purchase intention | After you understand the production place label of the aforementioned food, do you agree that you are willing to purchase the aforementioned food? | (4) |

Appendix B-1


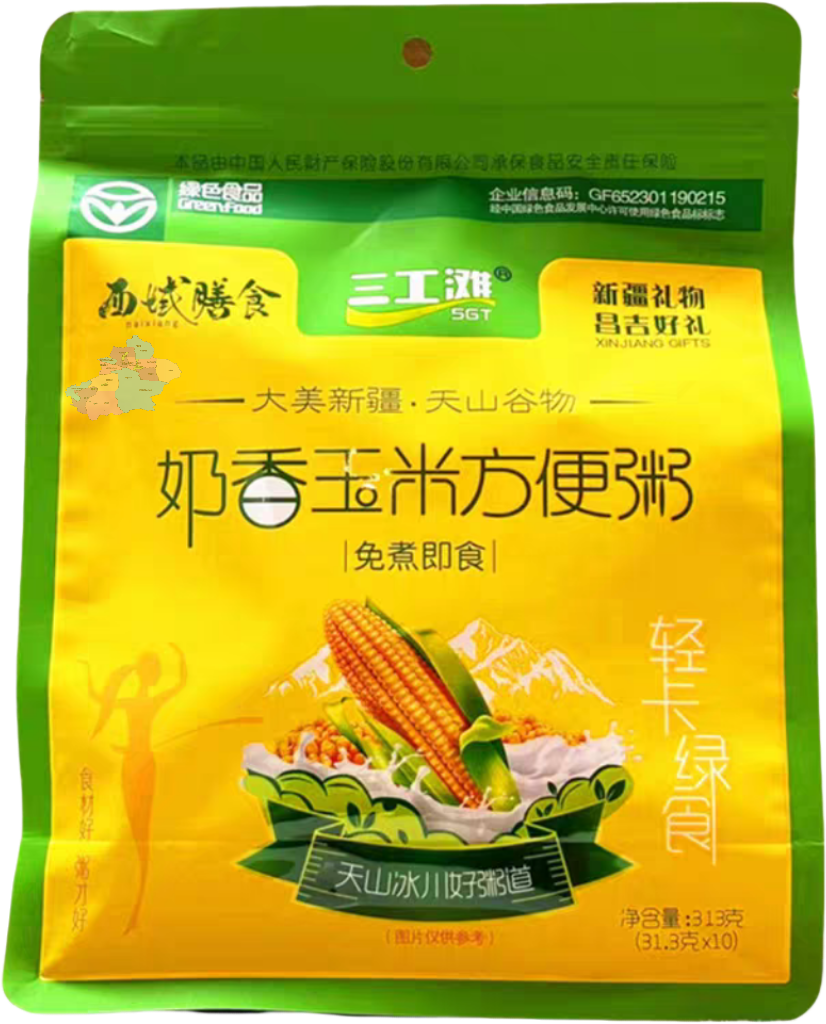


Product introduction: From the raw material level, its core raw material corn comes from the organic planting system. The growth process follows the natural ecological law and does not use chemically synthesized pesticides and fertilizers, which fundamentally ensures the purity and safety of raw materials. Organically grown corn, through scientific cultivation and ripening cycle, completely retains the original nutritional composition of corn, rich in dietary fiber, a variety of vitamins (such as vitamin B group, vitamin E) and minerals (such as potassium, magnesium, etc.), laying a solid foundation of nutritional value for readymade porridge. In processing, the product uses cutting-edge food processing technology to achieve a balance between convenience and nutrient retention. The processing process pays attention to the precise control of temperature and time, minimizes the damage to the nutritional components of corn, and ensures that the product meets the standard of ready-to-eat, meeting the demand of consumers for food convenience in modern fast-paced life. Packaging design also reflects the professional, the use of sealed, moisture-proof, fresh materials and technology, effectively extend the shelf life of the product, and in the process of storage and transportation can maintain the stability of food quality.

Appendix B-2


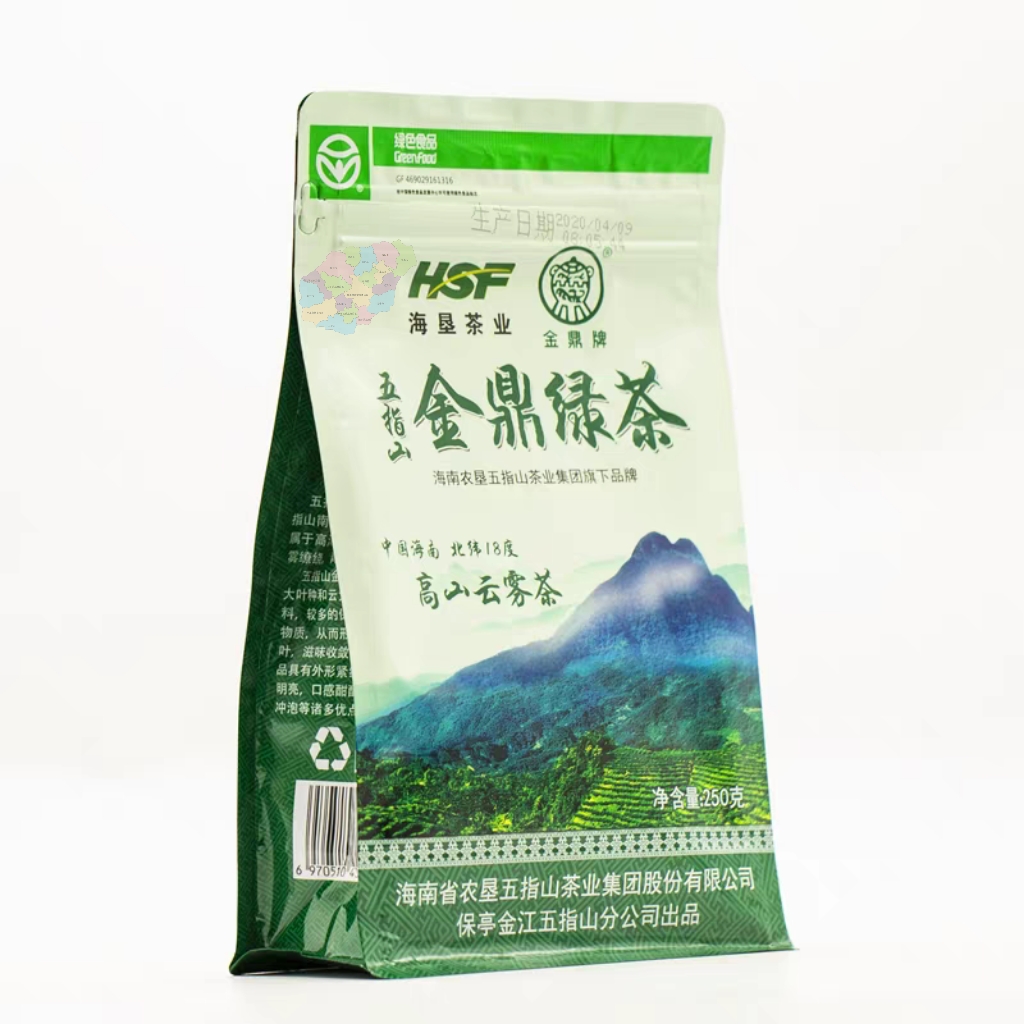


Product introduction: The raw materials of Jinding green tea are produced in the Yunwu tea garden of the high mountains. The planting system strictly follows the standards of organic agriculture, abandons chemical pesticides and synthetic fertilizers, and only uses natural biological control methods and organic fertilizer cultivation. Tea garden ecosystems achieve self-circulation through plant diversity layout, which significantly reduces exogenous chemical inputs. This planting mode not only ensures the purity of tea raw materials, but also effectively improves the diversity of soil microorganisms, providing stable ecological support for the synthesis of alkaloids, catechin polyphenols and theanine in tea. In the process, the product adopts the combination technology of low temperature steam killing and stone grinding. Steam greening temperature is controlled at 80-85℃, which can retain more heat-sensitive active ingredients in tea compared with traditional high-temperature greening. Stone grinding can avoid nutrient loss caused by high temperature friction through low-speed physical grinding, so that the particle size distribution of tea powder is more uniform, so as to improve the leaching efficiency of tea soup and achieve efficient release of nutrients.

Appendix B-3


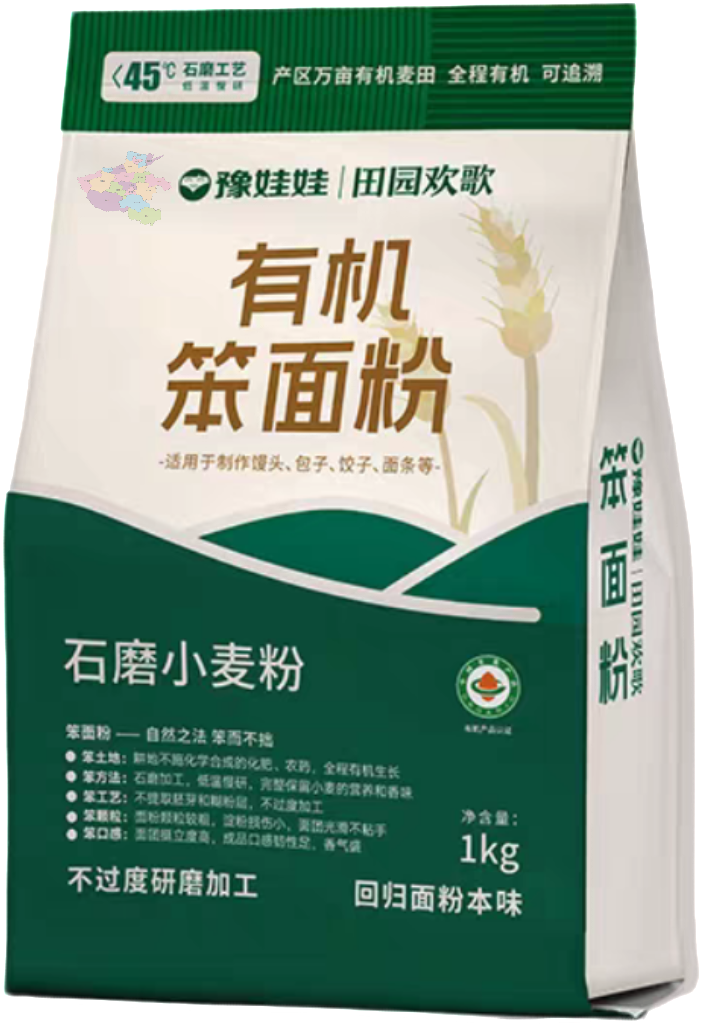


Product Introduction: The raw material of this flour comes from a strictly certified organic wheat farming base. The planting process follows the principles of organic agriculture and completely abandons chemical synthetic pesticides, fertilizers, growth regulators and other exogenous chemical inputs. The farm uses a rotation fallow system to enhance soil fertility by planting legumes for biological nitrogen fixation, while using biodiversity to build a natural pest control system. In the processing process, the traditional stone grinding technology is adopted, and the low speed grinding of the stone grinding makes the wheat endosperm broken into powder to avoid the destruction of nutrients by high temperature. Compared with modern steel grinding, stone grinding can completely retain key nutrients such as heat-sensitive vitamin E, wheat germ oil and gluten in wheat, while ensuring natural yellow color and rich flavor of flour. Its fine powder is moderate, which helps to evenly heat the baking process and achieve ideal baking expansion rate and taste toughness.

1. Liang B, Yang D, Tan F, Sun D, Li J. How psychological ownership over nutritional products affects purchase intentions of high-pressure working groups. Frontiers in Nutrition. 2024;11:1401035.

2. Asún R, Zúñiga C, Morales J-F. Design and validation of the revised Regional Identity Scale (RIS-2)/Diseño y validación de la Escala de Identidad Regional revisada (RIS-2). International Journal of Social Psychology. 2018;33(2):357-89.

3. Charton-Vachet F, Lombart C, Louis D. Impact of attitude towards a region on purchase intention of regional products: the mediating effects of perceived value and preference. International Journal of Retail & Distribution Management. 2020;48(7):707-25.

4. Yang D, Gui G, Yao Y, Ke X. Effect on consumers’ sustainable purchase intention of dietary supplement purine labeling. Frontiers in Nutrition. 2025;12:1526713.
